# Donor Proteinuria and Allograft Function in Kidney Transplantation: Short- and Long-Term Results From a Retrospective Cohort Study

**DOI:** 10.3389/ti.2023.11953

**Published:** 2023-12-14

**Authors:** Nicola Sariye Pollmann, Thomas Vogel, Caroline Pongs, Shadi Katou, Haluk Morgül, Philipp Houben, Dennis Görlich, Felicia Kneifel, Stefan Reuter, Lukas Pollmann, Andreas Pascher, Felix Becker

**Affiliations:** ^1^ Department of General, Visceral and Transplant Surgery, University Hospital Muenster, Muenster, Germany; ^2^ Institute of Biostatistics and Clinical Research, University Hospital Muenster, Muenster, Germany; ^3^ Department of Medicine D, Division of General Internal Medicine, Nephrology and Rheumatology, University Hospital Muenster, Muenster, Germany

**Keywords:** kidney transplantation, patient survival, graft survival, allocation, proteinuria

## Abstract

Donor proteinuria (DP) is a common but rarely evaluated aspect of today’s kidney transplant allocation process. While proteinuria after kidney transplantation is a risk factor for impaired graft function and survival, the long-term effects of DP in kidney transplantation have not yet been evaluated. Therefore, this study aims to investigate the impact of DP on the long-term outcome after kidney transplantation. A total of 587 patients were found to be eligible and were stratified into two groups: (1) those receiving a graft from a donor without proteinuria (DP−) and (2) those receiving a graft from a donor with proteinuria (DP+). At 36 months, there was no difference in the primary composite endpoint including graft loss and patient survival (log-rank test, *p* = 0.377). However, the analysis of DP+ subgroups showed a significant decrease in overall patient survival in the group with high DP (*p* = 0.017). DP did not adversely affect patient or graft survival over 36 months. Nevertheless, this work revealed a trend towards decreased overall survival of patients with severe proteinuria in the subgroup analysis. Therefore, the underlying results suggest caution in allocating kidneys from donors with high levels of proteinuria.

## Graphical Abstract

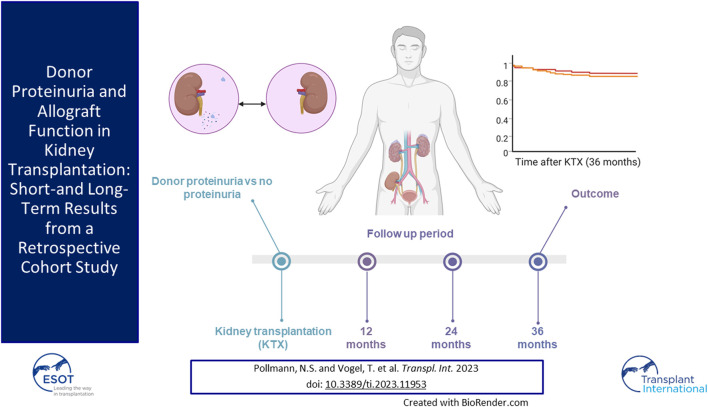

## Introduction

Donor shortage remains the cardinal problem of modern transplant medicine, especially in kidney transplantation (KTX). To address this ever-growing issue, multiple approaches have been taken to increase the donor pool, but the number of patients waiting for a suitable organ still exceeds the number of potential donors. Hence, the acceptance of marginal organs continues to increase [1]. Undoubtedly, these kidneys have a higher susceptibility to ischemia-reperfusion injury, combined with an undeniable risk of inferior long-term graft function [2]. These developments highlight the importance of a patient-based allocation with the characterization of harmful and harmless donor conditions.

Proteinuria is a common diagnosis after KTX and has been identified as an independent risk factor for inferior graft function and reduced graft survival after KTX [3–5]. Proteinuria can be diagnosed by a quantitative measurement of urine albumin or protein-to-creatinine ratio, as well as by albumin or protein excretion (PE) rate. In addition, a semiquantitative measurement with urine dipsticks can be used, as described by Kidney Disease: Improving Global Outcomes (KDIGO) and Chronic Kidney Disease (CKD) guidelines [6]. The prevalence of proteinuria in kidney transplant recipients ranges from 7.5% to 45% [7]. However, proteinuria is much more prevalent in organ donors, with low- and high-grade proteinuria occurring in 35.1% and 74.1% of allocated kidneys, respectively [8]. Yet, there are no official guidelines regarding donor proteinuria (DP) in kidney allocation, and the long-term impact of DP as an independent risk factor has not yet been validated. In consequence, DP can influence allocation decisions, with the inherent risk of declining suitable organs. This is especially important as most countries are experiencing a shortage of organ donors, resulting in long waiting times for patients on transplant lists. Therefore, declining a potentially suitable organ is negligent. On the contrary, proteinuria may indicate chronic kidney disease [9], and in kidney recipients, proteinuria is associated with reduced graft function and impaired 5 years graft survival. Patients with proteinuria have a survival rate of only 69%, compared to 93% for patients without proteinuria [10]. In kidney recipients with proteinuria, a decreased overall survival was observed compared to KTX recipients without proteinuria [11]. Additionally, patients with proteinuria have a 2.45-times increased risk of a cardiovascular event, such as ischemic heart disease, cerebrovascular disease, or peripheral vascular disease [7]. Multiple risk factors for posttransplant proteinuria have recently been defined, including a female donor, a male recipient, patients with acute rejection, donor age, and donor cardiovascular death [12, 13]. However, the effect of DP on proteinuria in the recipient has not yet been evaluated. This study aims to analyze the impact of DP on long-term (36 months) outcomes after KTX, specifically focusing on patient and graft survival.

## Materials and Methods

### Study Design and Study Population

**FIGURE 1:**
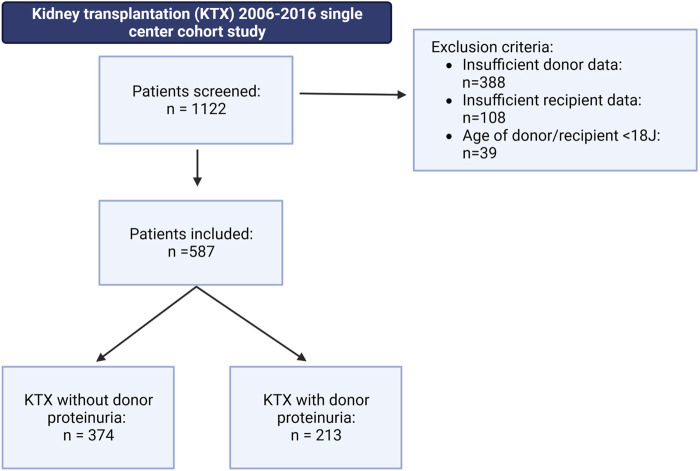
Patient selection within the underlying retrospective cohort study, including a 36 months follow-up. A total of 587 patients met the following inclusion criteria: kidney transplantation after deceased donor donation; donor or recipient age above 18 years; and complete donor and recipient dataset. Patients were stratified into two groups: (1) patients receiving a graft from a donor with proteinuria (DP+) and (2) patients receiving a graft from a donor without proteinuria (DP−).

This study was conducted as a retrospective single-center cohort study with follow-up of 36 months. All patients who underwent deceased-donor KTX at the University Hospital Münster, Germany, between 2006 and 2016 were screened for inclusion. Children under the age of 18, recipients of combined organ transplants, and recipients of living donations were excluded from this study. A total of 1,122 patients were initially screened, of whom 535 were excluded due to missing donor or recipient data or meeting the exclusion criteria (Figure 1). The remaining 587 eligible patients who met the inclusion criteria (deceased donation, age >18, complete dataset for recipient and donor) were further stratified into two groups: (1) patients who received a graft from a donor with proteinuria (DP+) and (2) patients who received a graft from a donor without proteinuria (DP−). Additionally, a subgroup analysis of the DP+ group was conducted, including four grades of DP severity: (+), +, ++, and +++. The study was conducted in accordance with the ethical principles outlined in the Declaration of Helsinki. The local ethics committee approved the study (Ethik-Kommission der Ärztekammer Westfalen-Lippe und Westfälischen Wilhelms-Universität, permit number: 2021-283-f-S). Written informed consent was not required as the study was a retrospective chart review. All data used in the final analysis were de-identified.

### Patient Cohort and Outcome Parameters

All grafts were procured on behalf of Eurotransplant (ET), and only grafts procured from donors after brain death were used. Donor characteristics were obtained from the Eurotransplant Network Information System (ENIS). Donor characteristics included age, sex, body mass index (BMI), cardiopulmonary resuscitation (CPR) and duration of CPR (in minutes), presence of comorbidities (hypertension, smoking, or diabetes mellitus), ischemia time, kidney donor risk index (KDRI), and kidney donor profile index (KDPI). In addition, the donor variables included extended criteria donor (ECD) status, which was defined as age ≥60 years or 50–59 years with at least two of the following conditions: a history of hypertension, a serum creatinine (sCr) level of 1.5 mg/dL, and a cerebrovascular cause of death. Other variables considered were use of vasopressors during donor evaluation, length of stay in the intensive care unit prior to donation, highest and most recent (at time of procurement) sCr levels (in µmol/L) during donor evaluation, cytomegalovirus (CMV) status, and human leukocyte antigen (HLA) mismatch. Complete donor urine findings were analyzed, including urine leukocytes, urine epithelial cells, urine bacteria, urine casts, urine erythrocytes, urine glucose, and the presence of proteinuria, measured by semiquantitative dipstick analysis. Recipient data were collected retrospectively from a prospective clinical database. Demographic recipient variables included age, sex, dialysis vintage, history of hypertension, and the reason for end-stage kidney disease (ESKD).

### Outcome Measures

Blood and urine samples were collected at various time points during the routine follow-up. Samples were taken immediately after the postoperative period, as well as at 3 (baseline), 6, 12, 24, and 36 months after KTX. The primary endpoint was a composite endpoint (event-free survival) that included graft loss and patient survival. It was estimated using the Kaplan-Meier methodology and compared using log-rank testing. Graft loss was defined as the need to reinitiate dialysis. Secondary outcome parameters included renal function, as measured by the estimated glomerular filtration rate (eGFR; mL/h/1.73 kg^2^, estimated using the Chronic Kidney Disease Epidemiology Collaboration (CKD EPI) formula)), PE per day (mg/d), and urine protein/creatinine ratio (UPCR; mg/g creatinine). Other outcome measures included primary non-function (PNF, defined as the need for continued dialysis within 90 days after KTX), DGF (defined as any need for dialysis within the first week after KTX), biopsy-proven acute rejection, new onset of diabetes after transplantation, and cardiovascular events (including myocardial infarction, angina pectoris, coronary artery revascularization, or congestive heart failure) after transplantation.

### Statistical Analysis

Normally distributed continuous variables are shown as the mean with standard deviation (SD), and not normally distributed continuous variables are presented as the median with interquartile range (IQR). Groups were compared using Student’s t-test for normally distributed data, Mann-Whitney U test for not normally distributed data, and Fisher’s exact test for categorical variables. The Kolmogorov-Smirnov test was used to analyze the distribution of continuous variables. Recipient kidney function parameters were analyzed using a mixed model for repeated measurements. The data for the variables UPCR and PE were logarithmically transformed before analysis. Comparisons of serum and urine parameters within each group were performed using a one-way analysis of variance (ANOVA). Additionally, within each time point, the DP+ group was compared to the DP− group. All *p*-values were adjusted using the Holm-Šídák method. Results are presented as the median and a 95% confidence interval. The probability of event-free survival, which includes patient survival and the probability of graft loss, was estimated using the Kaplan-Meier methodology, and all three endpoints were compared using the log-rank test (for *p*-values ≤0.05). Cox proportional hazards regression models were fitted to determine the influence of donor variables (proteinuria, age, cold ischemia time, CPR, sCr at procurement, hypertension, diabetes mellitus) on event-free survival, patient survival, graft loss, as well as reduced renal function (transformed to a dichotomous endpoint of eGFR </> 30 mL/h/1.73 kg). Hazard ratios (HR) and 95% confidence intervals (CI) were calculated. All statistical analyses and graphics were performed using IBM SPSS® Statistics 24 for Windows (IBM Corporation, Somers, NY, USA) and GraphPad Prism 10 software for Windows (GraphPad Software, CA, USA).

## Results

**FIGURE 2:**
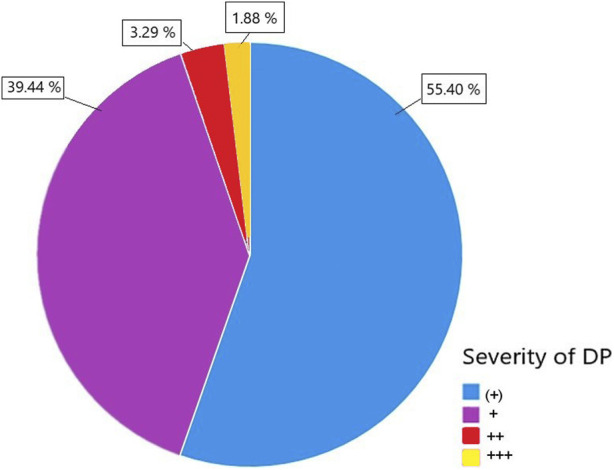
The severity of donor proteinuria (DP) was categorized into four levels: DP ^(+)^, DP^+^, DP^++^, and DP^+++^, specified by semiquantitative urine measurement.

A total of 587 patients met the inclusion criteria. This cohort was further stratified based on the presence of proteinuria in the donor. Out of the total patients, 213 (36.3%) received a DP+ graft, while 374 patients (63.7%) received a DP− organ (Figure 1). Within the DP+ cohort, the majority had low grade proteinuria (55.4%) followed by mild grade proteinuria (39.4%). Only a small fraction had moderate proteinuria (3.3%) or high-grade proteinuria (1.9%), as indicated by semiquantitative measurement (Figure 2).

Both groups showed similar demographic donor characteristics (Table 1). However, DP− donors had a slightly higher BMI (26.0 vs. 27.0, *p* = 0.012) and higher sCr level at the time of procurement (0.760 μmol/L vs. 0.555 μmol/L, *p* = 0.007) (Table 1). In addition, the frequency of ECD donors was significantly higher in the DP+ cohort (57.75%) compared to the DP− cohort (41.71%) (Table 1) (*p* < 0.001). Moreover, DP+ donors had significantly lower diuresis during donor evaluation (DP+: 129.2 mL/h, DP−: 166.7 mL/h) (*p* < 0.001). When analyzing urine parameters, the frequency of positive findings for urine leukocytes, urine casts, urine erythrocytes, and urine glucose was significantly higher in the DP+ cohort (Table 1). There were no significant differences regarding baseline demographic recipient characteristics between the DP+ and DP− groups (Table 2).

**TABLE 1:** Donor characteristics.

Variable	DP − *n* = 374	DP + *n* = 213	*p*-value
Age (years, mean ± SD)	54.6 ± 14.8	55.7 ± 15.3	0.401^a^
Sex (n, % males)	181 (48.4)	108 (50.7)	0.607^b^
Body mass index (kg/m^2^, median (IQR))	26.0 (24.0; 28.0)	27.0 (24.0; 30.0)	**0.012** ^c^
Serum Creatinine at procurement (µmol/l median (IQR))	70.70 (5.00, 97.20)	79.60 (54.80, 122.45)	**0.007** ^c^
Cardiopulmonary resuscitation (n, %)	79 (21.1)	49 (23.0)	0.605^b^
Duration of cardiac arrest (min, median (IQR))	15.0 (10.0; 45.0)	20.0 (10.0; 55.0)	0.555^c^
Hypertension (n, %)	121 (32.4)	77 (36.1)	0.365^b^
Diabetes mellitus (n, %)	30 (8.0)	23 (10.8)	0.295^b^
Smoking (n, %)	144 (38.5)	89 (41.7)	0.483^b^
Cold ischemia time (h, median, (IQR))	10.1 (7.4; 13.4)	10.1 (7.5; 13.3)	0.923^c^
Warm ischemia time (min., median, (IQR))	35.0 (30.0; 40.0)	32.0 (28.0; 40.0)	0.860^c^
Kidney donor profile index (median, (IQR))	70.0 (49.0; 92.0)	72.0 (49.0 94.0)	0.128^c^
Kidney donor risk index (median, (IQR))	1.2 (1.0; 1.6)	1.2 (1.0; 1.8)	0.062^c^
Expanded criteria donors (n, %)	234 (41.7)	101 (57.7)	**<0.001** ^b^
Perioperative vasopressors (n, %)	2 (0.8)	3 (0.9)	0.359^b^
Time at intensive care unit prior to donation (days, median, (IQR))	3.0 (2.0; 6.0)	3.0 (2.0; 6.5)	0.428^a^
Diuresis prior to donation (ml/h, median (IQR))	166.7 (115.9; 229.0)	129.2 (91.6; 204.0)	<**0.0001** ^a^
Cytomegalovirus risk status^d^			0.534^e^
Low (*n,%*)	132 (35.3)	75 (35.2)	
Intermediate (*n, %*)	91 (24.3)	44 (20.7)	
High (*n, %*)	151 (40.4)	94 (44.1)	
Human leukocyte antigen mismatch^f^			0.837^e^
0 (n, %)	62 (16.6)	32 (15.0)	
1–3 (n, %)	197 (52.7)	117 (54.9)	
4–6 (n, %)	115 (30.7)	64 (30.0)	
Urine leukocytes (n, %)	79 (21.12)	69 (32.39)	**0.003** ^b^
Urine epithelial cells (n, %)	23 (6.15)	22 (10.32)	0.076^b^
Urine bacteria (n, %)	35 (9.36)	26 (12.21)	0.325^b^
Urine casts (n, %)	10 (2.67)	17 (7.81)	**0.007** ^b^
Urine erythrocytes (n, %)	127 (33.96)	110 (51.64)	**<0.001** ^b^
Urine glucose (n, %)	65 (17.4)	62 (29.1)	**<0.001** ^b^

Results are presented as mean ± standard deviation (SD), median with interquartile range (IQR) or relative frequency.

^a^ Student’s t-test.

^b^ Fisher’s exact test.

^c^ Mann-Whitney U test.

^d^ Cytomegalovirus (CMV) risk status based on donor (d) and recipient (r) status low = d-/r-, intermediate: d-/r+ or d+/r+, high: d+/r-.

^e^ Chi square test.

^f^ Number of cumulative human leukocyte antigen mismatches.

Significant values are highlighted bold for clarity.

**TABLE 2:** Recipient characteristics.

Variable	DP − *n* = 374	DP + *n* = 213	*p*-value
Age (mean ± SD)	56.42 ± 12.39	57.00 ± 12.27	0.585^a^
Gender (n, % males)	226 (60.4)	135 (63.4)	0.537^b^
Dialysis vintage (month, median, (IQR))	66.0 (37.5; 93.5)	73.0 (43.0; 93.0)	0.403^c^
Hypertension before Transplantation (n,%)	362 (96.8)	200 (93.9)	0.113^b^
Diagnosis of end stage renal disease (n,%)			0.313^d^
Glomerulonephritis	106 (28.3)	50 (23.5)	
Diabetic nephropathy	28 (7.5)	25 (11.7)	
Hypertensive nephropathy	7 (1.9)	6 (2.8)	
Obstructive nephropathy	6 (1.6)	1 (0.5)	
Fokal segmental glomerulosklerosis	38 (10.2)	25 (11.7)	
Interstitial nephritis	14 (3.7)	8 (3.8)	
Vasculitis	12 (3.2)	3 (1.4)	
Chronic pyelonephritis	10 (2.7)	7 (3.3)	
Alport syndrome	5 (1.3)	4 (1.9)	
Autosomal dominant polycystic kidney disease 2	54 (14.4)	30 (14.1)	
Benign Nephrosclerosis	3 (0.8)	2 (0.9)	
Other	28 (7.5)	16 (7.5)	

Results are presented as mean ± standard deviation (SD), median with interquartile range (IQR) or relative frequency.

^a^ Student’s t-test.

^b^ Fisher’s exact test.

^c^ Mann-Whitney U test.

^d^ Chi square test.

**FIGURE 3:**
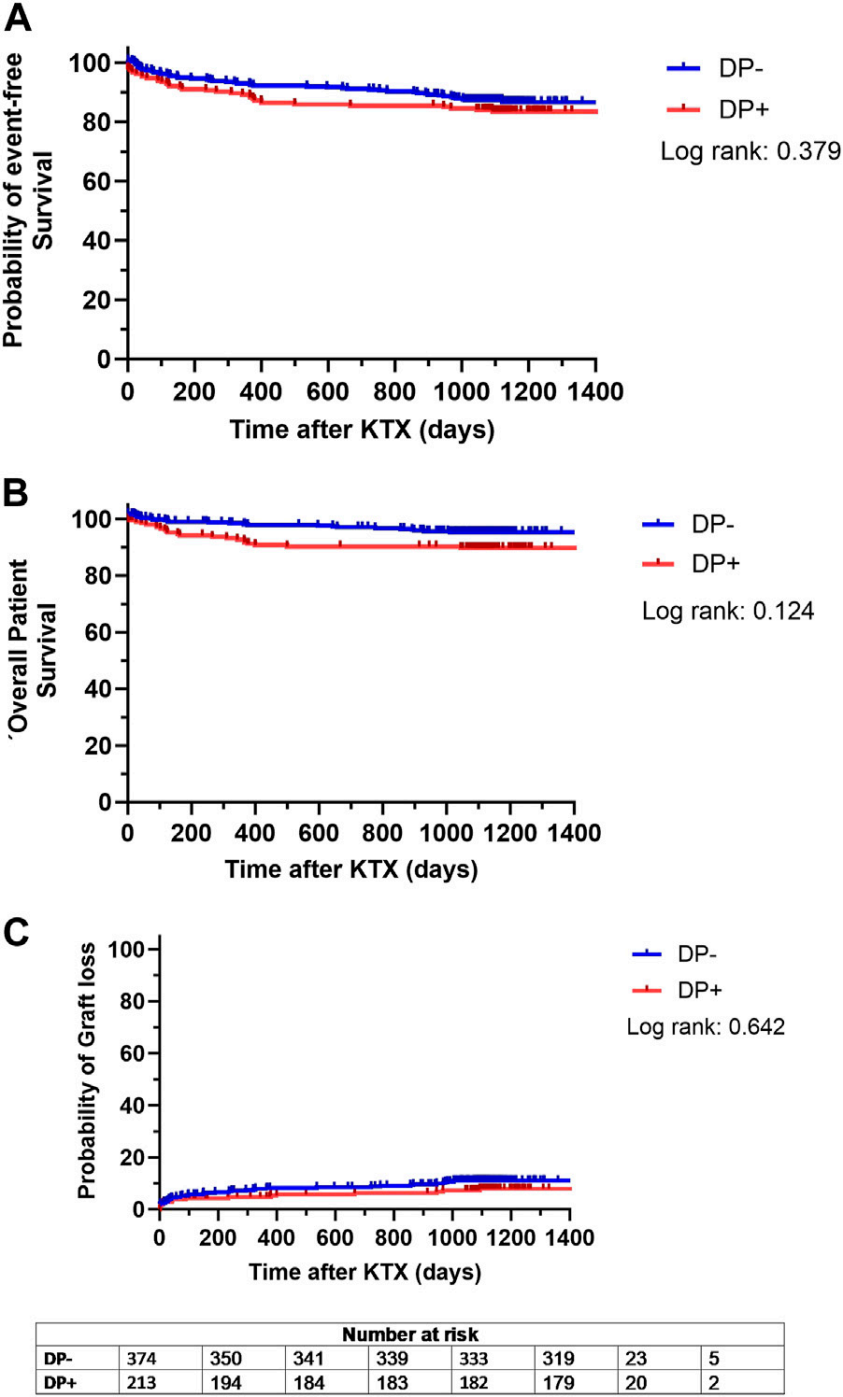
Kaplan Meier analysis for probability of **(A)** event-free survival, defined as combined patient and graft survival, **(B)** overall patient survival and **(C)** probability of graft loss separated for patients receiving a graft from a donor with proteinuria (DP+) and patients receiving a graft from a donor without proteinuria (DP−). Survival rates of DP+ (red lines) and DP− (blue lines) recipients following kidney transplantation (KTX) were estimated by Kaplan-Meier methodology and compared by log-rank test.

The combined endpoint of patient and graft survival, specifically the probability of event-free survival, did not differ significantly between both groups (DP+: 83.5% event-free survival; DP−: 85.5% event-free survival; *p* = 0.379) (Figure 3A). This indicates that DP did not negatively affect long term outcomes after KTX. In addition, patient survival was comparable (*p* = 0.124), with 89.8% for DP+ patients and 93.3% for DP− recipients (Figure 3B). There was an equally low probability of graft loss in both cohorts, with 9.0% in the DP− group and 7.9% in the DP+ group (*p* = 0.642) (Figure 3C). Therefore, the results suggest that neither long-term patient survival, nor long-term graft loss was impaired by DP.

**FIGURE 4:**
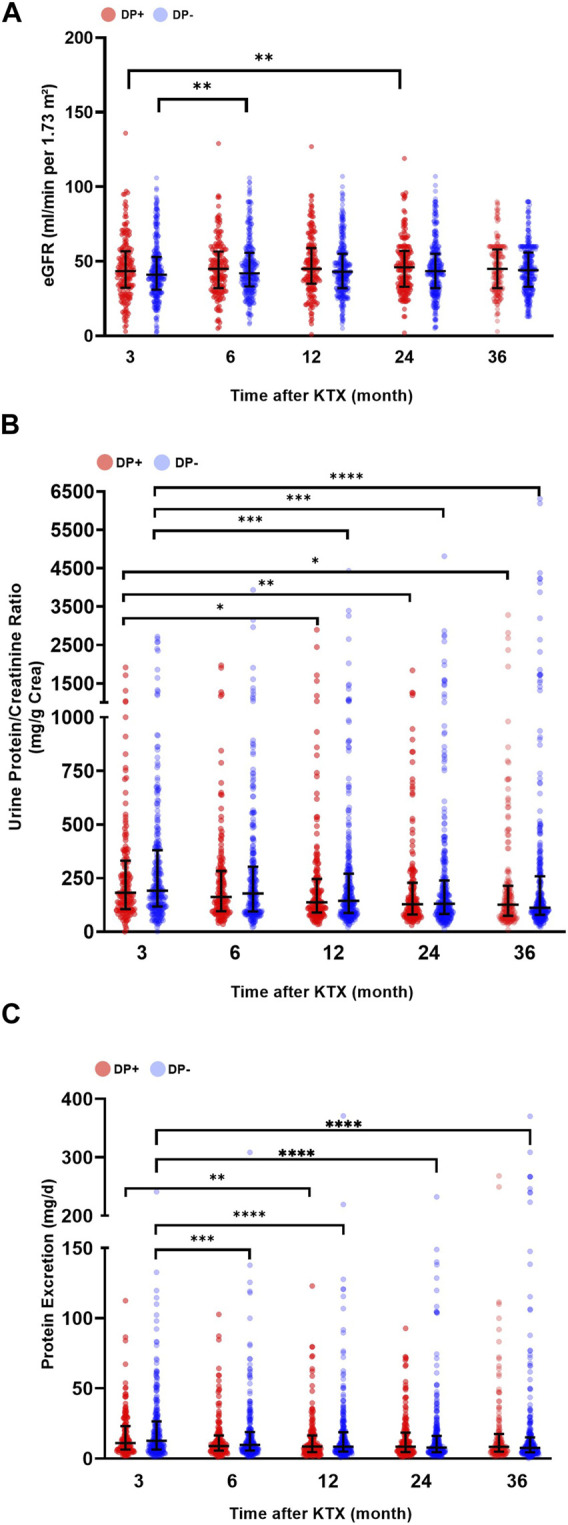
Post-transplant graft function and urine protein excretion. Serum and urine parameters of patients receiving a graft from a donor with proteinuria (DP+) and patients receiving a graft from a donor without proteinuria (DP−). **(A)** Estimated glomerular filtration rate (eGFR mL/min/1,73 m^2^), **(B)** urine protein/creatinine ratio (mg/g creatinine; UPCR), and **(C)** urine protein excretion (mg/d; PE) after kidney transplantation (KTX). Comparisons of serum and urine parameters within each group were performed using a one-way analysis of variance (ANOVA). The data for the variables UPCR and PE were logarithmically transformed before analysis. Within each time point, the DP+ group was compared to the DP− group. All *p*-values were adjusted using the Holm-Šídák method. A *p*-value less than 0.05 was considered statistically significant, *p ≤ 0.05; **p ≤ 0.01; ***p ≤ 0.001; ****p ≤ 0.0001.

When analyzing post-transplant renal function, it was observed that the DP− and DP+ cohorts had similar eGFR at 3, 6, 12, 24, and 36 months after KTX (Figure 4A). However, longitudinal analysis within each cohort revealed a significantly higher eGFR (compared to baseline) 6 months after KTX in the DP− cohort (*p* = 0.005). Additionally, in the DP+ cohort, the eGFR at 24 months after KTX was significantly higher than at the 3 months baseline (*p* = 0.010) (Figure 4).

The comparison of post-transplant UPCR revealed decreasing values for both groups over time. Both the DP− and DP+ cohorts showed a significant decrease in UPCR at 12, 24, and 36 months after KTX compared to the 3 months baseline (Figure 4B). In addition, the overall urine PE in the DP− group was significantly lower at 6, 12, 24, and 36 months compared to the baseline at 3 months. In contrast, the DP+ group showed a significant decrease in urine PE 12 months after KTX compared to the 3 months baseline. Overall, the DP+ group showed lower values for PE and UPCR compared to the DP− group, but these differences were not statistically significant (Figure 4C).

Analysis of secondary endpoints showed no significant difference between the DP+ and DP− cohorts for the incidence of DGF, PNF, biopsy-proven rejection, new onset of diabetes after transplantation, or cardiovascular events after transplantation (Table 3).

**TABLE 3:** Secondary endpoints.

	DP − *n* = 374	DP + *n* = 213	*p*-value
Primary nonfunction (n, %)	21 (5.6)	11 (6.6)	0.718^a^
Delayed graft function (n, %)	87 (23.3)	52 (24.4)	0.763^a^
Biopsy proven acute rejection (n, %)	183 (48.9)	101 (47.4)	0.731^a^
New onset of diabetes after transplantation (n, %)	44 (11.8)	29 (13.6)	0.603^a^
Cardiovascular event after transplantation (n, %)	37 (9.9)	24 (11.3)	0.673^a^

Results are presented as relative frequency.

^a^ Fisher’s exact test.

To explore independent donor-associated risk factors, univariate and multivariate Cox regression models were used for the following endpoints: event-free survival (including patient and graft survival), patient survival, graft survival, and marginal renal function (eGFR <30 mL/h/1.73 m^2^). Regarding event-free survival, both univariate and multivariate analyses showed no significant association with proteinuria, cold ischemia time, CPR, sCr at procurement, or diabetes mellitus (Table 4). However, donor age was found to be significantly associated with a reduced probability of event-free survival in both univariate analysis (HR: 1.05 [1.03–1.08], *p* < 0.001) and multivariate analysis (HR: 1.05 [1.03–1.08], *p* < 0.001) (Table 4). Donor age was also found to negatively affect patient survival (HR: 1.04 [1.01–1.06], *p* = 0.002) (Table 5), graft survival (HR: 1.04 [1.03–1.06], *p* < 0.001) (Table 6), and renal function (HR: 1.03 [1.02–1.05], *p* < 0.001) (Table 7), all in the multivariate analysis, respectively. In addition, hypertension was found to be associated with a reduced probability of event-free survival (HR: 1.92 [2.00–3.36], *p* = 0.022) in the univariate analysis (Table 4). It was also associated with reduced graft survival (HR: 1.62 [1.06–2.5], *p* = 0.025) (Table 6) and marginal renal function (HR: 1.60 [1.06–2.40], *p* = 0.025) (Table 7).

**TABLE 4:** Cox regression model of event-free survival.

	Univariate		Multivariate	
Donor characteristics	*p*-Value	HR (95% Cl)	*p*-Value	HR (95% Cl)
Proteinuria (yes/no)	0.651	0.87 (0.48–1.58)	0.554	0.83 (0.45–1.53)
Age (years)	**<0.001**	1.05 (1.03–1.08)	**<0.001**	1.05 (1.03–1.08)
Cold ischemia time (hours)	0.988	1.00 (0.93–1.07)	0.316	1.04 (0.97–1.11)
Cardiopulmonary resuscitation (yes/no)	0.539	0.80 (0.39–1.64)	0.846	1.08 (0.51–2.28)
Last serum creatinine (µmol/L)	0.302	1.00 (0.99–1.00)	0.364	1.00 (0.99–1.00)
Hypertension (yes/no)	**0.022**	1.92 (1.10–3.36)	0.471	1.25 (0.69–2.26)
Diabetes mellitus (yes/no)	0.063	2.05 (0.96–4.38)	0.460	1.35 (0.61–2.99)

Hazard ratios (HR) and 95% confidence intervals (CI).

Significant values are highlighted bold for clarity.

**TABLE 5:** Cox regression model of patient survival.

	Univariate		Multivariate	
Donor characteristics	*p*-Value	HR (95% Cl)	*p*-Value	HR (95% Cl)
Proteinuria (yes/no)	0.130	1.57 (0.88–2.83)	0.229	1.44 (0.80–2.62)
Age (years)	**0.001**	1.04 (1.02–1.06)	**0.002**	1.04 (1.01–1.06)
Cold ischemia time (hours)	0.499	1.03 (0.95–1.10)	0.141	1.06 (0.98–1.14)
Cardiopulmonary resuscitation (yes/no)	0.296	0.65 (0.29–1.46)	0.421	0.71 (0.30–1.65)
Last serum creatinine (µmol/L)	0.327	1.00 (1.99–1.01)	0.257	1.00 (1.00–1.01)
Hypertension (yes/no)	0.063	1.74 (0.97–3.13)	0.396	1.31 (0.71–2.42)
Diabetes mellitus (yes/no)	0.552	1.33 (0.52–3.36)	0.942	0.97 (0.37–2.53)

Hazard ratios (HR) and 95% confidence intervals (CI).

Significant values are highlighted bold for clarity.

**TABLE 6:** Cox regression model of graft survival.

	Univariate		Multivariate	
Donor characteristics	*p*-Value	HR (95% Cl)	*p*-Value	HR (95% Cl)
Proteinuria (yes/no)	0.378	1.21 (0.79–1.86)	0.588	1.13 (0.73–1.75)
Age (years)	<**0.001**	1.04 (1.02–1.06)	**<0.001**	1.04 (1.03–1.06)
Cold ischemia time (per hour)	0.269	1.03 (0.98–1.08)	**0.018**	1.06 (1.01–1.12)
Cardiopulmonary resuscitation (yes/no)	0.189	0.68 (0.34–1.21)	0.471	0.80 (0.44–1.46)
Last serum creatinine (µmol/L)	0.760	1.00 (1.00–1.01)	0.555	1.00 (1.00–1.01)
Hypertension (yes/no)	**0.025**	1.62 (1.06–2.48)	0.563	1.14 (0.73–1.79)
Diabetes mellitus (yes/no)	0.182	1.54 (0.82–2.90)	0.705	1.14 (0.59–2.20)

Hazard ratios (HR) and 95% confidence intervals (CI).

Significant values are highlighted bold for clarity.

**TABLE 7:** Cox regression model of renal function.

	Univariate		Multivariate	
Donor characteristics	*p*-Value	HR (95% Cl)	*p*-Value	HR (95% Cl)
Proteinuria (yes/no)	0.378	1.21 (0.79–1.86)	0.588	1.13 (0.73–1.75)
Age (years)	<**0.001**	1.04 (1.02–1.06)	**<0.001**	1.04 (1.03–1.06)
Cold ischemia time (per hour)	0.269	1.03 (0.98–1.08)	**0.018**	1.06 (1.01–1.12)
Cardiopulmonary resuscitation (yes/no)	0.189	0.68 (0.39–1.21)	0.471	0.80 (0.44–1.46)
Last serum creatinine (µmol/L)	0.760	1.00 (1.00–1.00)	0.555	1.00 (1.00–1.01)
Hypertension (yes/no)	**0.025**	1.62 (1.06–2.48)	0.563	1.14 (0.73–1.79)
Diabetes mellitus (yes/no)	0.182	1.54 (0.82–2.90)	0.705	1.14 (0.59–2.20)

Hazard ratios (HR) and 95% confidence intervals (CI).

Significant values are highlighted bold for clarity.

**FIGURE 5:**
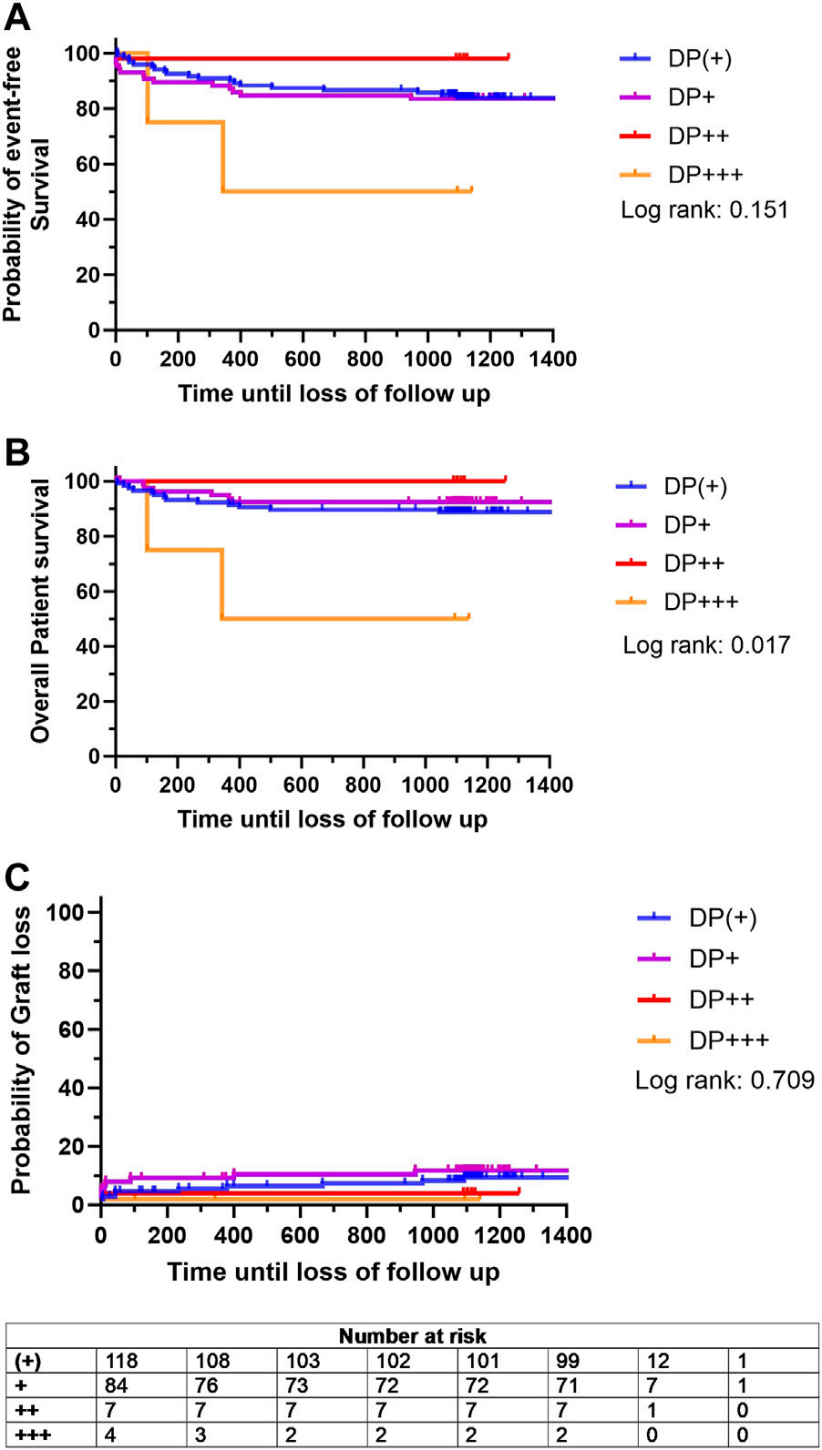
Kaplan Meier analysis for probability of **(A)** event-free survival, defined as combined patient and graft survival, **(B)** overall patient survival and **(C)** probability of graft loss stratified based on the degree donor proteinuria (DP): DP ^(+)^ (green line), DP^+^ (purple line), DP^++^ (red line), and DP^+++^ (yellow line). Survival rates were estimated by Kaplan-Meier methodology and compared by log-rank test.

To further investigate whether the severity of DP would impact the outcome after KTX, a subgroup analysis was conducted within the DP+ group. When stratified for DP severity, the probability of event-free survival did not differ significantly among the DP ^(+)^, DP^+^, and DP^++^ groups (83.7%, 84.5%, and 100%, respectively) (Figure 5A). However, the DP^+++^ cohort showed a tendency towards decreased event-free survival compared to the other subgroups, although this difference remained statistically insignificant (50.0%, *p* = 0.151) (Figure 5A). In addition, the overall patient survival of the DP ^(+)^, DP^+^, and DP^++^ cohorts were comparable (89.6%, 92.4%, and 100%) (Figure 5B). A significant decrease in overall patient survival was observed in the DP^+++^ cohort compared to the other subgroups (50.0%, *p* = 0.017) (Figure 5B). The probability of graft loss was equally low in the DP ^(+)^, DP^+^, DP^++^, and DP^+++^ groups (*p* = 0.709) (Figure 5C).

With respect to the excretory renal parameters, a similar range was observed within the subgroups over time. The DP^+++^ cohort showed an overall trend of reduced eGFR. However, this reduction was only significant 24 months after KTX (*p* < 0.0001). In addition, PE was increased in the DP^+++^ group at three and 6 months after KTX (536.7 mg/gr Cr and median = 443.2 mg/gr Cr, respectively), but this increase did not reach statistical significance (Supplementary Figure S1). The analysis of UPCR showed relatively low parameters in the DP ^(+)^, DP^+^, and DP^++^ groups.

## Discussion

Proteinuria is a well-described feature after KTX, but its impact on graft and patient outcomes remains uncertain, making it an undefined factor in the kidney allocation process. Therefore, this study investigated long-term outcomes in KTX patients, stratified based on donors with and without proteinuria. Additionally, the underlying investigation aimed to evaluate DP as a potential risk factor for post-transplant proteinuria. Proteinuria is known to be a prognostic factor for poor long-term outcomes, including reduced patient and graft survival as well as an increased risk for cardiovascular events after KTX [7]. This study established that within a 36 months post-transplant follow-up period, DP was not associated with impaired patient or graft survival or impaired graft function. Our results affirm the previous findings of Kuhn et al. [8], who demonstrated that there was no effect of DP in KTX on graft survival or function within 12 months after surgery [8].

It has been thoroughly established that KTX is associated with improved survival, reduced morbidity, and increased quality of life when compared to long-term dialysis [14]. However, KTX is facing an ever-growing obstacle due to the declining number of donated organs. Thus, the shortage of donors demands the optimal utilization of every potentially suitable organ. Critical assessment of donor organ quality in deceased donor KTX includes evaluating urine findings. Among the challenges of analyzing urine findings in deceased donors is that pathological findings may not always indicate preexisting chronic kidney disease. This is also true for proteinuria in donors, which could be caused by trauma, intense exercise, dehydration, fever, or a urinary tract infection. At the same time, DP could be the result of a glomerular disorder, including focal segmental glomerulosclerosis, glomerulonephritis, diabetic or hypertensive nephropathy, and vasculitis [15]. Thus, when DP is included in the decision-making process of donor selection, the involved surgeons and nephrologists are at risk of either declining a suitable graft or accepting a graft with structural kidney damage. To address this dilemma, this study aimed to investigate the impact of DP on outcomes after KTX. For this purpose, 578 patients were enrolled in the study and closely monitored at our interdisciplinary KTX clinic. As this investigation was conducted at a single center, we were able to utilize nearly complete datasets for analyzing the long-term effects.

Both donors and recipients showed similar baseline demographic variables in the DP+ and DP− groups. Nevertheless, some differences were observed with less favorable features, including a higher rate of ECD grafts in the DP− donor group compared to the DP+ cohort. On the other hand, DP− donors showed a lower BMI and a higher eGFR prior to KTX compared to DP+ donors. The higher rate of ECD kidneys in the DP− cohort may have influenced the results of this study in favor of DP+ donors. However, since the KDPI and KDRI were similar in both groups and the eGFR rates were initially higher in DP− grafts, the difference may be less significant.

DP was not associated with impaired event-free survival and did not affect patient survival or the likelihood of graft loss. This demonstrates that DP did not negatively affect long term outcomes after KTX and thus, transplantation of grafts from donors with low-grade DP is safe with regard to short- and long-term outcomes. In both the univariate and multivariate analyses of donor characteristics, DP was not identified as a risk factor for any of the three defined endpoints.

As indicated by both UPCR and PE parameters, DP was not associated with post-transplant proteinuria over time after KTX. Both UPCR and PE parameters concordantly decreased within both experimental groups over the observed time. However, the increment in PE development was stronger in the DP− group compared to the DP+ group. Nevertheless, DP was not associated with high PE and UPCR values. In fact, the DP+ group showed even smaller PE and UPCR values compared to the DP− group. Therefore, this study indicated that DP was not associated with post-transplant proteinuria after KTX. The analysis of eGFR in the DP+ group over 36 months showed significantly higher values at 12 months after KTX compared to the 3 months time point.

Concordantly with findings of previous studies, donor age was associated with a higher risk of impaired overall patient survival, death-censored graft survival, and event-free survival within this investigation [16, 17]. In addition, the underlying study confirmed that donor hypertension is a risk factor for impaired graft survival and event-free survival [18, 19]. According to current literature, these results show that higher donor ages and hypertension negatively affect overall patient survival [20]. Therefore, the validity of the underlying results can be assumed.

The semiquantitative measurement of proteinuria within this study, using urine dipsticks, undoubtedly represents one main limitation of this investigation. It is important to note that with proper quantification in the donor, one could better extrapolate how a high degree of UPE would impact post-transplant graft function. Furthermore, urine dipstick measurements should be interpreted with caution because they correlate poorly with the albumin-to-creatinine ratio (ACR), have low sensitivity and specificity, and have not yet been evaluated in renoprotective randomized controlled trials [19]. On the other hand, this method is used for kidney allocation, and specialized transplant surgeons select suitable kidney grafts based on semiquantitative measurements of proteinuria. Despite its drawback, dipstick analysis correlates with end-stage renal disease and is a widely used screening parameter [19]. In addition, this study confirmed recent findings on DP in KTX, even for a long-term period of up to 36 months after transplantation. Similar to the previous investigation on DP KTX [8], the underlying study did not investigate DP as a combined risk factor in ECD kidneys. Therefore, future investigations should outline the possibility that DP could be a combined risk factor in elderly donors (e.g., ≥60 years) with diabetes and nicotine abuse. In addition, it would be helpful to correlate the degree of DP with pre-transplant or implantation biopsies to better test the hypothesis that severe DP is indicative of structural kidney damage in the donor.

Interestingly, the subgroup analysis of the DP+ group revealed a significant decrease in overall patient survival in the group with high DP (*p* = 0.017). The results indicate an adverse effect of high-grade DP on long-term patient survival and are further supported by the observation of a reduced eGFR in the DP group. As stated earlier, proteinuria after KTX is a well-known risk factor for impaired graft survival [10]. This study indicates that donors with high levels of proteinuria might have an impact on the long-term graft performance in KTX.

However, our findings suggest that low-grade DP does not imply a risk of long-term complications or an influence on graft survival. Nevertheless, we highlight the need for further research on DP with respect to high proteinuria in quantitative urine measurements. Additionally, there is a need for further testing of donors at risk, particularly those who are older (e.g., ≥60 years) or have diabetes.

## Conclusion

In conclusion, this retrospective cohort study of 587 patients investigated the impact of DP from a 36 months perspective. No effect on patient or graft survival was observed in low-grade DP. This indicates that transplantation of grafts from donors with low-grade DP is safe with regard to short- and long-term outcomes. Nevertheless, differences in the secondary endpoint analysis revealed a trend towards decreased patient survival and eGFR values in DP+ patients, especially in subgroups with severe proteinuria. Therefore, the underlying results suggest caution when allocating kidneys from donors with high levels of proteinuria.

## Data Availability

The raw data supporting the conclusion of this article will be made available by the authors, without undue reservation.

## Supplementary Material

SUPPLEMENTARY FIGURE S1 Post-transplant graft function and urine protein excretion in patients receiving a graft from a donor with proteinuria (DP). Subgroups were stratified based on the degree of DP: (+), +, ++, and +++. **(A)** Estimated glomerular filtration rate (eGFR mL/min/1,73 m^2^), **(B)** urine protein/creatinine ratio (mg/g creatinine; UPCR), and **(C)** urine protein excretion (mg/d; PE) after kidney transplantation (KTX). Comparisons of serum and urine parameters within each group were performed using a one-way analysis of variance (ANOVA). The data for the variables UPCR and PE were logarithmically transformed before analysis. All *p*-values were adjusted using the Holm-Šídák method. A *p*-value less than 0.05 was considered statistically significant, **p* ≤ 0.05; ***p* ≤ 0.01; ****p* ≤ 0.001; *****p* ≤ 0.0001.
