# Supplementary figures and images for: Donor Proteinuria and Allograft Function in Kidney Transplantation: Short- and Long-Term Results From a Retrospective Cohort Study
Source: Transpl Int. 2023 Dec 14;36:11953. doi: 10.3389/ti.2023.11953 (PMC10754218; doi:10.3389/ti.2023.11953)

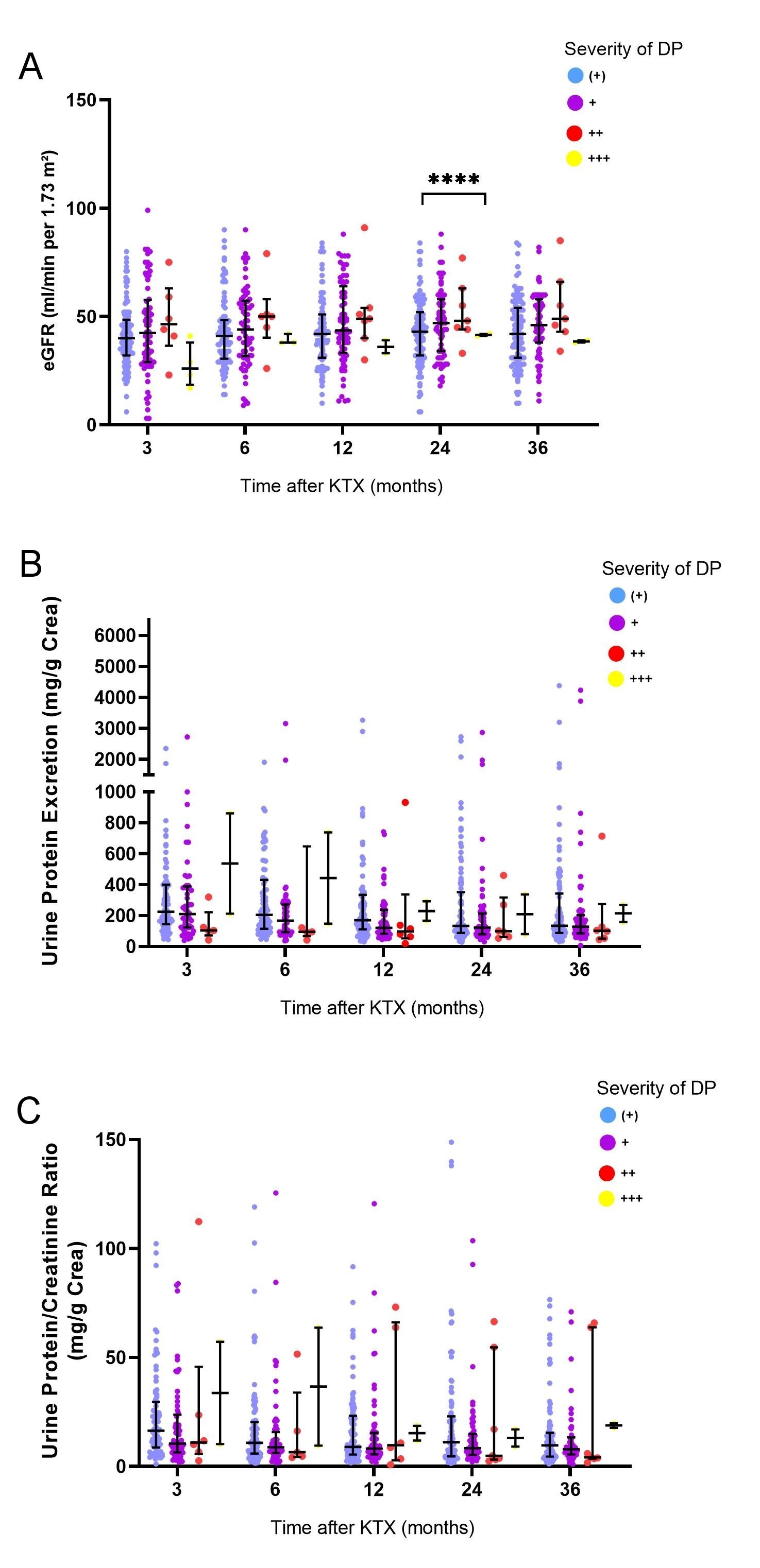

Supplement: Supplementary file 1 [file Image1.jpg]
